# The up-regulation of miR-21 by gastrodin to promote the angiogenesis ability of human umbilical vein endothelial cells by activating the signaling pathway of PI3K/Akt

**DOI:** 10.1080/21655979.2021.1964895

**Published:** 2021-08-23

**Authors:** Jianli Wang, Minghua Wu

**Affiliations:** a Department Of Neurology, Affiliated Hospital Of Nanjing University Of Chinese Medicine, Nanjing, China; b Department Of Neurology, Jiangsu Province Hospital Of Chinese Medicine, Nanjing, China

**Keywords:** Gastrodin, huvecs, angiogenesis, pi3k/akt signal, miR-21

## Abstract

Studies have shown that gastrodin has a protective effect on blood vessels. The purpose of this study was to investigate the influence of gastrodin on the angiogenesis ability of human umbilical vein endothelial cells (HUVECs) and its mechanism. We found that treatment of HUVECs with 10 µM and 25 µM gastrodin, and Vascular endothelial growth factor (VEGF) significantly upregulated the miR-21 expression in the cells. Meanwhile, gastrodin significantly increased the cell proliferation, migration and tube formation ability of HUVECs and increased the expression of MMP-2 and MMP-9 mRNA. In addition, gastrodin promoted the phosphorylation level of PI3K/Akt protein. However, down-regulating the miR-21 expression reduced the promoting effect of gastrodin on the HUVECs angiogenesis. In conclusion, gastrodin activates the PI3K/Akt pathway by up-regulating the miR-21 expression and promotes the HUVECs angiogenesis.

## 1. Introduction

Angiogenesis, which plays a vital role in various biological processes, is defined as the formation of new blood vessels in the human body [1]. Angiogenesis is critical for the treatment of many diseases, including stroke, myocardial infarction and cardiovascular disease. The absence of blood vessels in metabolically active tissues may inhibit the repair of damage or other essential functions [2]. As we all know, high disability, high incidence, and high recurrence rate are the characteristics of cerebral infarction, an acute ischemic cerebrovascular disease, accounting for 60–80% of the total strokes [3]. Cerebral ischemia will lead to irreversible degeneration and necrosis of a large number of neurons, causing severe neurological deficits [4]. In facts, promoting the angiogenesis of ischemic cerebral to increase the number of new collateral circulation will increase blood supply and improve ischemic brain function [5]. Vascular endothelial growth factor (VEGF), an early angiogenic cytokine after injury, is an important factor in angiogenesis, and can increase the proliferation and migration of endothelial cells, as well as promoting angiogenesis [6]. However, the production and effect of VEGF in the body are limited, and it cannot be quickly nor effectively solve the body damage or disease caused by insufficient blood supply. Therefore, the identification and application of specific compounds that may induce neovascularization may help solve the clinical problems of ischemia-related diseases.

Gastrodin is the main active ingredient in *Gastrodia*, which belongs to the genus Gastrodin (Orchidaceae) [7]. Traditional Chinese medicine believes that *gastrodia* can be used to treat cardiovascular and cerebrovascular diseases, such as cardiac hypertrophy, fibrosis, migraine, stroke and hypertension [8]. Clinically, gastrodin is used to reduce systolic blood pressure and pulse pressure, and the treat senile hypertension, with analgesic and sedative effects [9]. It has been reported that gastrodin exerts antioxidant and anti-inflammatory functions through suppressing pro-inflammatory factors and scavenging oxygen free radicals [10]. And there are some reports about the protective effect of gastrodin on blood vessels, Lin et al. found that gastrodin can avoid the damage caused by tert-butyl hydroperoxide (TBHP) to human umbilical vein endothelial cells (HUVECs) [11]. Chen et al. found that gastrodin prevents the damage of HUVECs induced by homocysteine by increasing the activity of Nrf2/ARE and PI3K/Akt/eNOS pathways [12].

On the other hand, microRNA (miRNA) is a type of naturally existed short non-coding RNA whose function is to suppress the expression of specific genes encoding target proteins after transcription [13]. In recent years, emerging evidence has confirmed the key role of miRNA in the vascular and endothelial cell biology [14]. miR-21, an oncogenic miRNA, has also been confirmed that it exert influence in tumor blood vessels [15]. An et al. found that miR-21, which is highly expressed in adipose-derived stem cell exosomes, can promote endothelial cell vascularization [16]. However, the mechanism of how gastrodin regulates the angiogenesis of HUVECs by miRNA is still unclear. Therefore, in this study, we first determined the regulatory effect of gastrodin on the angiogenesis of HUVECs, and then through cell experiments to further explore the specific mechanism of how gastrodin influencing the function of HUVECs by regulating miR-21. Our study provides a theoretical basis for the clinical treatment of vascular-related diseases with gastrodin.

## 2. Materials and methods

### 2.1 Cell culture and processing

HUVECs were obtained from the American Type Culture Collection (ATCC). The cells were cultured in DMEM medium containing 100 mg/ml streptomycin 100 U/ml penicillin, and 10% fetal bovine serum (FBS, all obtained from Gibco, USA), and incubated in a 5% CO_2_ humidified atmosphere at 37°C.

HUVECs were treated with 10 ng/mL VEGF (VEGF group), 10 µM gastrodin (GSTD-10 μM group), 25 μM gastrodin (GSTD-25 μM group). In addition, the HUVECs were transfected with NC inhibitor and miR-21 inhibitor using lipo 3000 (Thermo, USA). MiR-21 inhibitor and NC inhibitor were obtained from GenePharm (Shanghai, China).

### 2.2 Quantitative RT-PCR (qRT-PCR)

Total RNA was extracted with TRizol reagent (Invitrogen, USA). After determining the RNA purity and concentration, cDNA was converted following the instruction of random primer reverse transcription kit (Thermo, USA). Then cDNA (2 µl) was used in a 20 µl qPCR reaction including 5 µM of each primer and 10 µl SYBR GREEN PCR Master Mix (TaKaRa, Japan). The reactions were performed on a CFX 96 Touch Real-Time PCR Detection System (Bio-Rad) with the cycling condition: 95°C for 10 min and 40 cycles of 95°C for 10 s, and 58°C for 30 s. GAPDH and U6 were internal reference controls, and this experiment should be repeated for 6 times. Relative gene expression was calculated using the 2^−ΔΔCt^ method. The primer sequences were designed and synthesized by Sangon Biotech (Shanghai, China), and the primer sequences are shown in Table 1.

**Table 1.** Quantitative primers

RNA	Sequences (5ʹ to 3ʹ)
miR-21	F: 5ʹ- GCTTATCAGACTGATGTTG
	R: 5ʹ- GAACATGTCTGCGTATCTC
MMP-2	F: 5ʹ- AGCGAGTGGATGCCGCCTTTAA
	R: 5ʹ- CATTCCAGGCATCTGCGATGAG
MMP-9	F: 5ʹ- GCCACTACTGTGCCTTTGAGTC
	R: 5ʹ- CCCTCAGAGAATCGCCAGTACT
U6	F: 5ʹ- CTCGCTTCGGCAGCACAT
	R: 5ʹ- TTTGCGTGTCATCCTTGCG
GAPDH	F: 5ʹ- GTCTCCTCTGACTTCAACAGCG
	R: 5ʹ- ACCACCCTGTTGCTGTAGCCAA

### 2.3 Detection of 5-Ethynyl-2ʹ-deoxyuridine (EdU)

The cells were transferred into a plate with 24 wells, stained strictly followed by the EdU staining kit instruction (Thermo, USA). We used a fluorescence microscope (model: FM-600, Shanghai Pudan Optical Instrument Co., Ltd.), and randomly chose 6–10 fields to observe, recorded positive cells number after sealing. EdU labeling rate (%) = positive cells number/ (positive cells number + negative cells number) ×100%.

### 2.4 MTT assay

After the treatment, cells were transferred into 96-well plates with a density of 1000 cells/well. A total of 20 μL MTT solution (5 mg/mL; Sigma, USA) was added into each well, and the cells continued to culture for 4 h. At 24 h, 48 h, and 72 h after culture, DMSO of 150 μL was added, shaken for 15 minutes, and then the optical density (OD) of a wavelength of 570 nm of each well was measured by a Microplate reader (Thermo Fisher Scientific, USA).

### 2.5 Scratch test

First, we used a marker pen to draw a horizontal line with a ruler on the back of the 6-well plate, inoculated the cells into the six-well plate, and treated them separately to cultivate until confluence. A 10 μL sterile pipette tip was used to make a draw perpendicular to the ruler. PBS was used to wash and remove the suspended cells and debris. Fresh serum-free medium was added, and the cells were cultured for 24 hours and then shot with an inverted microscope, and the results of the experiment were recorded. Image J software was used to calculate the scratch area.

### 2.6 Tube formation test

Matrigel (Biosciences, USA) of 100 μL was transferred into each well of a 24-well plate, which was later incubated at 37°C for 30 minutes. After different treatments with HUVECs, the cells were re-suspended in DMEM without FBS and inoculated at a concentration of 1 × 10^5^ cells/well. After 6 hours, the formation of capillary structures was evaluated under an optical microscope, and scanning and quantification were performed as well.

### 2.7 Western blot

Proteins were isolated from cells using a RIPA lysis buffer (Sigma, USA), and the protein concentration was determined by the BCA kit (Beyotime, China). After the SDS-PAGE separation, the proteins were transferred to the PVDF membrane (Millipore, USA). The membrane was blocked with 5% skimmed milk for 1 h at room temperature, and then incubated with primary antibody PI3K (#4249, Cell Signaling Technology), p-PI3K (ab278545, Abcam), Akt (#4685, Cell Signaling Technology), p-Akt (#4060, Cell Signaling Technology) and GAPDH (10,494-1-AP, Proteinintech) overnight at 4°C. Subsequently, the membrane was washed for 3 times, followed by incubation with the corresponding secondary antibody (Abcam) 1 h at room temperature. After the membrane was washed for three times, chemiluminescence reagent was added to develop the protein and it was placed in the gel imaging system to collect images. Image J software was performed to analyze the gray level of protein bands. The relative protein expression was calculated while selecting GAPDH as an internal reference.

### 2.8 Data processing and analysis

SPSS 26.0 was employed for data analyses. The independent sample t-tests or one-way analysis of variance (ANOVA) were applied to evaluate difference between two groups or multiple groups. The results were expressed as mean ± standard deviation (SD). p < 0.05 represented that the there was a significant difference.

## 3. Results

Studies have reported that gastrodin has a protective effect on blood vessels. and miR-21 is also involved in regulating vascular endothelial cells. Therefore, the aim of this study was to investigate the functional and mechanistic effects of gastrodin on the angiogenic capacity of HUVECs. Our experiments also confirmed that gastrodin could upregulate miR-21 expression in HUVECs and promote angiogenesis in HUVECs by activating PI3K/Akt signaling pathway.

### 3.1 Gastrodin promotes the proliferation of HUVECs

**Figure 1.**
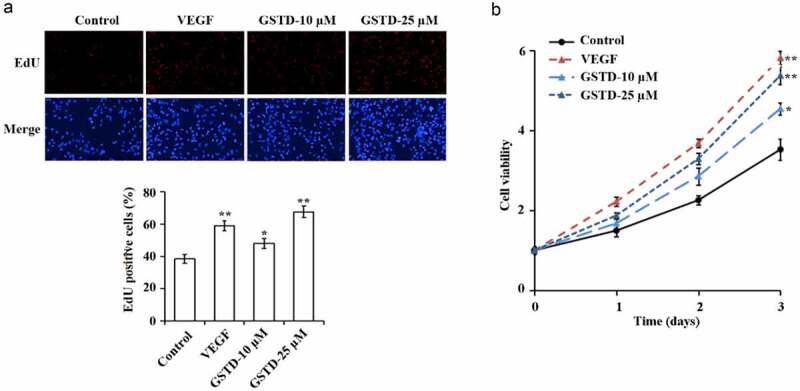
Gastrodin promotes the proliferation of HUVECs A: determination of cell proliferation using EdU; B: assessment of cell proliferation using MTT. * p < 0.05; ** p < 0.01. GSTD, gastrodin

The detection results of EdU and MTT have showed (Figure 1(a-b)) that in comparison with the Control group, the cell proliferation levels of the VEGF group, GSTD-10 µM group, and GSTD-25 µM group were notably higher. And GSTD was concentration-dependent, the higher the concentration, the higher the cell proliferation level.

### 3.2 Gastrodin promotes the migration and tube formation of HUVECs

**Figure 2.**
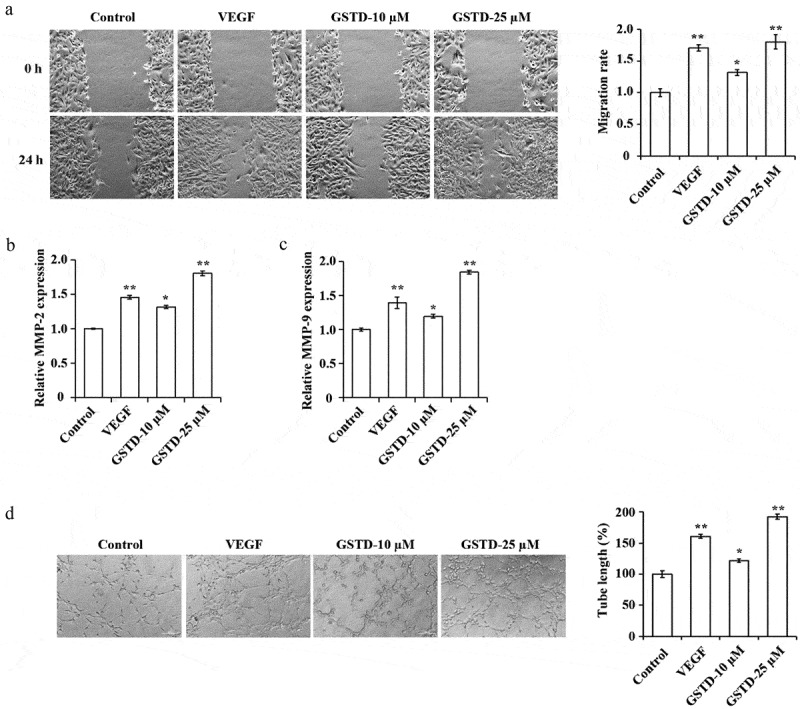
Gastrodin promotes the migration and tube formation of HUVECs A: determination of cell migration ability using scratch test; B: assessment of the expression of MMP-2 using qRT-PCR; C: assessment of the expression of MMP-9 using qRT-PCR; D: evaluation of cell tube formation ability using tube formation test. * p < 0.05; ** p < 0.01. GSTD, gastrodin

The HUVECs migration ability and angiogenesis ability of each group were tested, and the results showed (Figure 2(a-d)) that when comparing to the Control group, the tube formation and cell migration ability of the VEGF group, GSTD-10 µM group, and GSTD-25 µM group were notably higher, the MMP-2 and MMP-9 expression levels increased distinctly. GSTD was concentration-dependent, the higher the concentration, the stronger the tube formation and cell migration ability, the higher the MMP-2 and MMP-9 expression levels.

### 3.3 Gastrodin upregulates miR-21 expression

**Figure 3.**
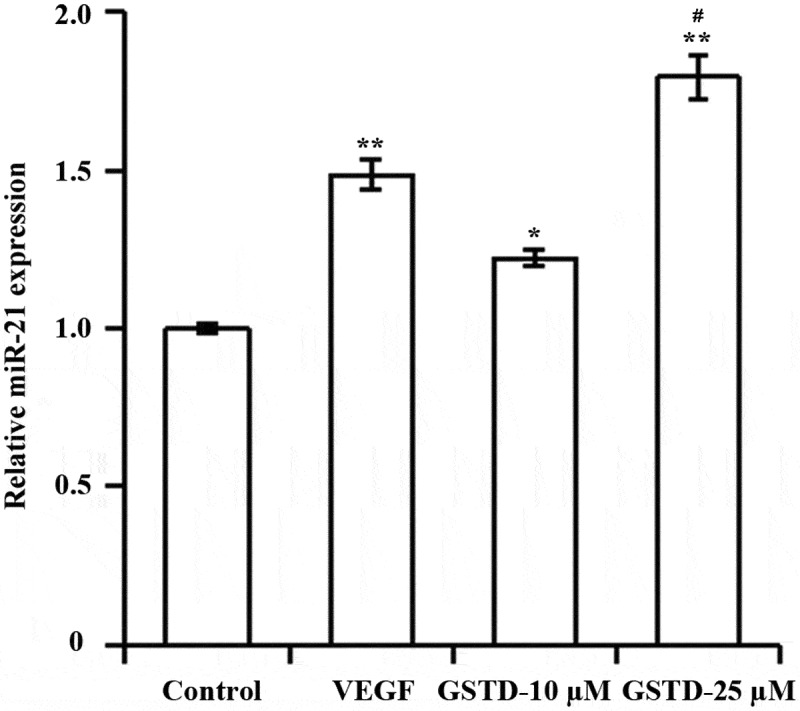
Gastrodin upregulates miR-21 expressionDetermination of miR-21 expression level using qRT-PCR; * p < 0.05; ** p < 0.01 vs. control group. # p < 0.05 vs. GSTD-10 µM group. GSTD, gastrodin

As shown by qRT-PCR (Figure 3), both VEGF and GSTD significantly upregulated the expression of miR-21 in HUVECs compared with Control group. And miR-21 was significantly higher in GSTD-25 µM group than in GSTD-10 µM group.

### 3.4 Gastrodin affects the proliferation, migration and tube formation ability of HUVECs via miR-21

**Figure 4.**
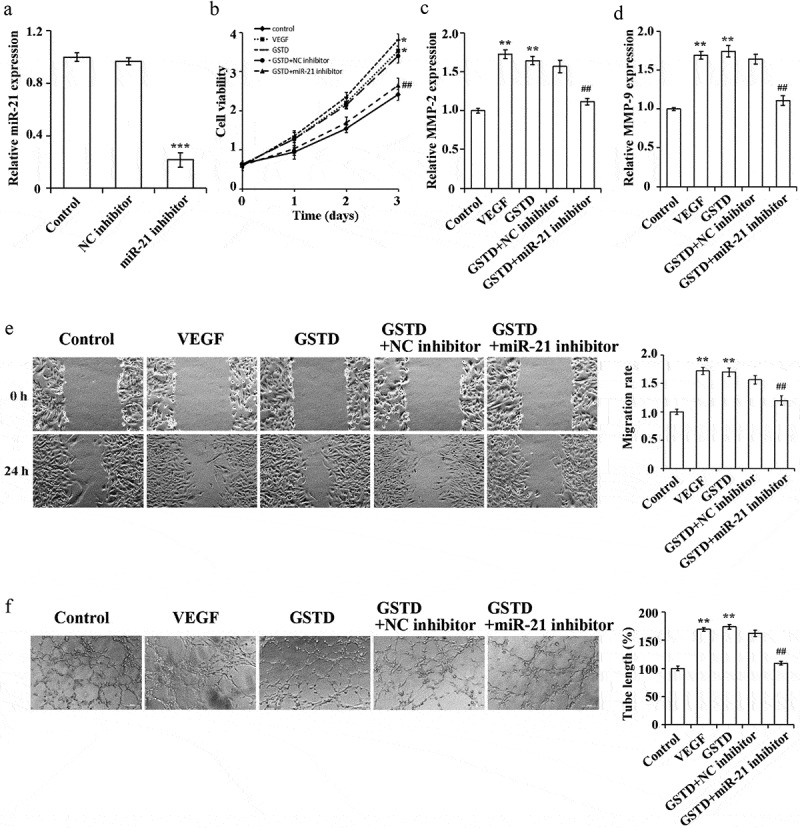
Gastrodin affects the proliferation, migration and tube formation ability of HUVECs via miR-21 A: determination of miR-21 expression level using qRT-PCR, *** represented comparison with NC inhibitor group, and p < 0.001; B: assessment of cell proliferation using MTT; C: determination of MMP-2 expression using qRT-PCR; D: determination of MMP-9 expression using qRT-PCR; E: assessment of the migration ability using scratch test; F: assessment of the tube forming ability using tube formation test. ** p < 0.01; ## represented comparing to GSTD+NC inhibitor group, and p < 0.01. GSTD, gastrodin

In order to further determine the mechanism of how does GSTD regulate miR-21, we interfered with the miR-21 expression in HUVECs. qRT-PCR result has verified the transfection efficiency. When comparing to the NC inhibitor group, miR-21 inhibitor can inhibit the miR-21 expression in HUVECs (Figure 4a), p < 0.05). When comparing to the Control group, the tube formation ability, cell proliferation rate, and cell migration ability in the VEGF group and GSTD group were notably higher, and the MMP-2 and MMP-9 expression levels were notably higher. Interfering with the miR-21 expression can suppress the promoting effect of GSTD on the cell proliferation rate, migration ability and tube formation ability of HUVECs. At the same time, the MMP-2 and MMP-9 expression levels were also notably lower (Figure 4(b-f)). These results confirmed that by up-regulating the miR-21 expression, GSTD can promote the tube formation, cell migration and proliferation of HUVECs.

### 3.5 Gastrodin activates the PI3K/Akt signaling pathway of HUVECs

**Figure 5.**
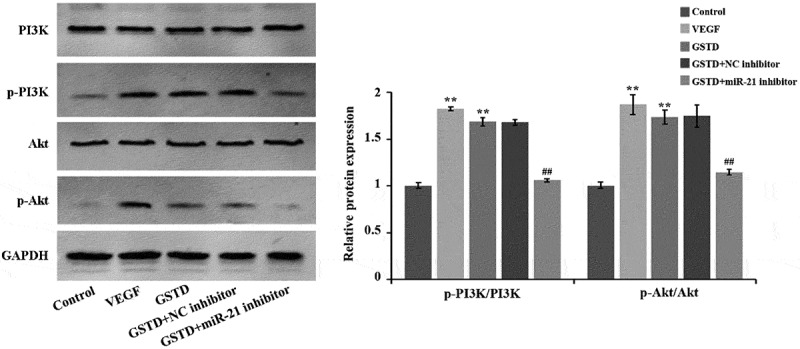
Gastrodin activates the PI3K/Akt signaling pathway of HUVECs evaluation of the protein expression levels of PI3K, p-PI3K, Akt and p-Akt using western blot. **p < 0.01; ## represented comparing to GSTD+NC inhibitor group, and p < 0.01. GSTD, gastrodin

Western blot results (Figure 5) showed that when comparing to the Control group, the protein levels of p-PI3K and p-Akt in the VEGF and GSTD groups were notably higher, and the p-PI3K/PI3K and p-Akt/Akt ratio was notably higher. When comparing to the GSTD+NC inhibitor group, the p-Akt and p-PI3K of the GSTD+miR-21 inhibitor group were notably reduced in the protein levels, and the p-Akt/Akt and p-PI3K/PI3K ratio was distinctly reduced. All these results indicate that GSTD can activate the signaling pathway of PI3K/Akt by regulating miR-21.

## 4. Discussion

Angiogenesis is a key process of embryonic development, tissue growth and wound healing [1]. In the process of angiogenesis, the endothelial cells located in the inner wall of the vasculature respond to growth signals, such as basic Fibroblast Growth Factor (bFGF), VEGF, cytokines or nitric oxide, thereby proliferating and migrating to form new blood vessels [17]. Pathologically speaking, angiogenesis can promote the healing of inflammation and abnormal wound, meanwhile, it can accelerate the development and progression of vascular disease and tumor as well [18]. This study also confirmed that VEGF can induce the proliferation, migration and tube formation of HUVECs. Besides, from previous studies, it was found that gastrodin can suppress the apoptosis induced by preeclampsia by regulating the TLR4/NF-κB of HTR/SVneo cells [19]. Liu et al. found that gastrodin antagonized the up-regulation of stress signals from the endoplasmic reticulum by activating the Nrf2 pathway, including the expression of GRP78, CHOP, and phosphorylated eIF2α, reducing osteoporosis in rats caused by glucocorticoids [20]. Zhuo et al. found that adding gastrodin to the cell model MRC-5 of chronic obstructive pulmonary disease can suppress the LPS-induced activation of p38/JNK and NF-κB pathways by down-regulating the miR-103 expression [21]. It can be seen that gastrodin plays a positive effect in the disease treating, and exert a certain protective effect on the body. In this study, it was found that gastrodin can notably increase the proliferation, migration, tube formation, and MMP-9 and MMP-2 expression of HUVECs. With the increasing gastrodin concentration, the promotion effect would be better, which is consistent with the function of HUVECs induced by VEGF, suggesting that gastrodin can promote the angiogenesis of HUVECs though its specific mechanism of action has not been clarified.

On the other hand, miRNAs have been identified as epigenetic regulators essential for gene expression in regulating cell life activities [22]. In cell biology, the role of miRNAs is multidimensional. MiR-21 is a mature cancer-related miRNA (ie oncomiR), since miR-21 is up-regulated in a variety of tumors, which can promote the growth and invasion of many cancer cells [23]. Studies have pointed out that miR-21 induces and promotes wound re-epithelialization, wound contraction, collagen deposition and inflammation healing after skin injury [24]. In this experiment, gastrodin can notably up-regulate the miR-21 expression in HUVECs, which suggests that gastrodin exerts its biological function through regulating miR-21. When inhibiting the miR-21 level while treating with gastrodin, it can be observed that the effect of gastrodin on promoting angiogenesis of HUVECs was weakened, indicating that miR-21 is the key information transmission medium for gastrodin to exert its biological function. Similarly, Hu et al. found that exosomes from human umbilical cord blood can accelerate skin wound healing through angiogenesis and fibroblast function mediated by miR-21-3p [25]. Dai et al. found that in the tumor, andrographolide inhibits the miR-21-5p expression and down-regulates the activity of the TIMP3 pathway to inhibit angiogenesis [26]. Based on the evidence above, it can be seen that the miR-21 expression has a positive correlation with the cell angiogenesis ability.

Besides, studies have shown that the signaling pathway of PI3K/Akt is important for cancer development, and essential for signal transduction in normal cells as well [27]. It has been acknowledged that this pathway plays key roles in many cell functions, including metabolism, migration, proliferation, angiogenesis, adhesion and invasion [28]. Lu et al. found that miR-21 inhibition can down-regulate the activity of PI3K/Akt/VEGF pathway dependent on PTEN in diabetic retinopathy, and inhibit the growth and angiogenesis of retinal vascular endothelial cells [29]. Liu et al. found that by activating PI3K/Akt pathway, miR-21-21 could promote the healing of fracture [30]. The results of this study showed that gastrodin can notably activate the PI3K/Akt pathway of HUVECs. Thus, it can be seen that gastrodin promotes the angiogenesis of HUVECs by activating the PI3K/Akt pathway and up-regulating the miR-21 expression.

## 5. Conclusion

In summary, gastrodin can increase the miR-21 expression in HUVECs, activate the signaling pathway of PI3K/Akt, and improve cell proliferation, migration and tube formation, suggesting that gastrodin can induce the formation of new blood vessels, which is helpful to solve the problem of treating the ischemia-related diseases clinically. However, the interactions between gastrodin and miR-21 and PI3K/Akt pathway remain unknown, so further research is needed to fully clarify the role of gastrodin to make it can be better used in clinical practice.
